# Sample holder development for *in-situ* EBAC mapping of the uniformity of thin-film conductivity

**DOI:** 10.1016/j.mex.2026.103892

**Published:** 2026-03-28

**Authors:** Isaac Appelquist Løge, Håkon Kristiansen, Nadia Shardt, Tore Sandnes Vehus

**Affiliations:** a Department of Chemical Engineering, Norwegian University of Science and Technology (NTNU), Sem Sælands vei 4 (Gløshaugen) 7491 Trondheim, Norway; b Technical University of Denmark, Department of Chemical Engineering, Søltofts Plads 228A, Kgs. Lyngby 2800, Denmark; c Department of Engineering Sciences, University of Agder (UiA), Universitetsveien 25 4630 Kristiansand, Norway

**Keywords:** Electron beam-induced current (EBIC), Electron beam absorbed current (EBAC), Thin-film conductivity, Titanium dioxide (TiO_2_), Nanoparticle networks, Film uniformity, Scanning electron microscopy (SEM)

## Abstract

Electron beam absorbed current (EBAC) microscopy can provide spatially resolved electrical information that conventional probe methods and local scanning probes often miss in nanoparticulate thin films. Here we present the development of a practical scanning electron microscopy (SEM)-based methodology to qualitatively assess conductivity uniformity and electrical continuity in thin films spanning two electrodes using TiO_2_ films as a model system. The approach combines a purpose-built insulating holder, readily made without clean-room and advanced lithography access, with embedded electrodes and a measurement configuration to visualize current pathways and identify electrically disconnected regions that may not be apparent from morphology alone. Line scans and 2D maps enable rapid screening of film quality, highlighting cracks, and poor particle connectivity. The workflow is designed for reproducibility and can be adapted to other semiconducting or weakly conducting thin films where microscale continuity is critical to device performance.

Fabricate a biasable dual-electrode SEM holder by embedding bent copper plates in an insulating resin body and machining a defined deposition channel (drilled to expose copper + resin in one plane), enabling repeatable electrode gaps and robust external connections for EBAC measurements.

Prepare thin films that reliably bridge the electrode gap by using a diluted, well-dispersed nanoparticle suspension (e.g., TiO_2_ in IPA) and controlled drop-casting into the channel; confirm continuous coverage using standard SEM imaging before electrical mapping.

Map conductivity uniformity in situ using EBAC (line scans and 2D maps under applied voltage) to rapidly locate conductive pathways and diagnose electrically disconnected regions that may appear morphologically continuous, providing a reproducible screening workflow adaptable to other weakly conducting thin films.

## Graphical abstract

**Figure:**
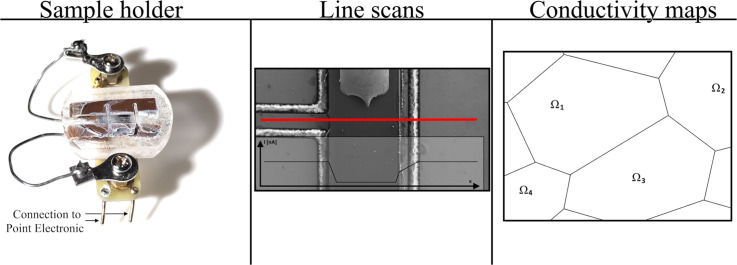

Specifications table

**Table:** 

Subject area	Engineering
More specific subject area	Thin-film characterization; SEM-based microscopy; conductivity mapping and film connectivity in nanoparticulate oxide films (TiO_2_)
Name of your method	EBAC line-scan and mapping for thin-film conductivity uniformity using a dual-electrode SEM sample holder
Name and reference of original method	*Electron beam-induced current (EBIC) microscopy as implemented in SEM for spatially resolved electrical measurements in semiconductors and functional materials* [1,2]*; adapted here for two-terminal thin-film continuity testing using a custom embedded-electrode holder.*
Resource availability	The method is described sufficiently in depth for reproduction.

## Background

Thin-film materials such as titanium dioxide (TiO_2_) are widely used in applications from photocatalysis to photovoltaics, where their electrical conductivity and uniformity can critically impact performance [3]. In nanoparticulate TiO_2_ films (e.g. in dye-sensitized solar cells or photocatalytic coatings), ensuring a continuous conductive network is vital for efficient charge transport [4]. However, measuring the local conductivity or connectivity of such thin films is challenging [5]. Traditional two- or four-point probe methods yield only a bulk-averaged sheet resistance and cannot resolve non-uniformities or isolated insulating regions within the film. Scanning probe techniques like conductive AFM can map local conductivity, but they are limited to very small areas and can suffer from tip-induced damage or poor repeatability. Advanced characterization methods such as transmission electron microscopy (TEM) and X-ray microscopy have also been used to probe electrical phenomena in oxides [6]. In their standard implementations, however, these methods do not automatically provide a simple, in situ map of lateral continuity. At the same time, important current-based variants have been demonstrated in recent years, including TEM/STEM beam-induced current imaging of resistive oxides [7] and X-ray beam-induced current approaches in scanning transmission X-ray microscopy [8]. These reports underscore the ongoing research in this field, but SEM-based workflows remain attractive because they are comparatively accessible, require minimal sample preparation, and can be coupled directly to routine imaging.

Using an electron beam to irradiate the sample and generate a mapping through the current offers a powerful alternative for spatially resolved electrical characterization of semiconducting or dielectric films and devices. Here, the current is collected through external circuitry and is recorded as a function of beam position [1,2]. In semiconductor junction studies, contrast often arises from separation of beam-induced carriers by built-in electric fields. This mode of operation is referred to as the electron beam induced current (EBIC) mode. If charge carriers are not induced, but instead the absorbed beam current is collected as it has passed through the sample, the mode refers to the electron-beam-absorbed current (EBAC) mode, in which image intensity depends on how efficiently the charge injected or removed by the beam is transported to the contacted electrodes [9,10]. Such absorbed-current modes have been used to map connectivity in metal interconnects, oxide ceramics, resistive switching oxides, and nanoparticle networks [6,[11], [12], [13]].

In this work, we describe the design of a specialized sample holder and experimental setup that allows *in-situ* current mapping of a thin film bridging two electrodes in an SEM. By applying an external bias across the film and scanning the electron beam across the sample, we visualize the continuous current path (or lack thereof) through the film. The method provides a spatial map or line-scan of conductivity, identifying any non-uniform regions, which could be caused by cracks, poor particle connectivity, or other defects that would impede current flow [5]. We also discuss the sample preparation steps (including slurry composition and deposition technique) required to obtain a uniform conductive film. This approach is general and can be applied to other semiconducting or conducting thin films to assess film continuity, complementing conventional electrical measurements. Compared with silicon wafers and patterned-electrode platforms used for nanoparticle network studies (e.g. [11]), the present approach offers lower-cost, cleanroom-free fabrication and rapid iteration of holder geometry; the trade-off is reduced flatness and geometric control of the surface, which we discuss explicitly in the limitations section.

## Method details

### Materials and thin-film preparation

**Fig. 1:**
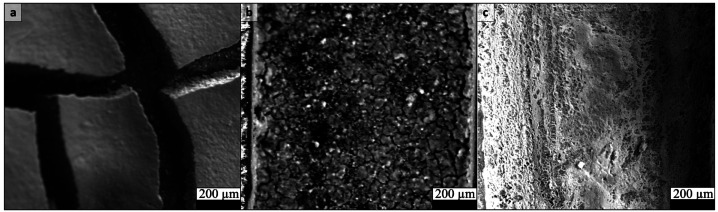
SEM images of drop-casted thin films of TiO_2_ with different mixing ratios and dispersion methods. (a) a 1:1 (wt:wt) TiO_2_–IPA mixture with no ultrasonication. (b) a 1:10 (wt:wt) TiO_2_–IPA mixture with no ultrasonication. (c) a 1:10 (wt:wt) TiO_2_–IPA mixture with 10 min of ultrasonication.

For our case study, TiO_2_ films were prepared on a custom sample holder (described below) that provides two separated electrode contacts. We used TiO_2_ nanoparticulate powder (P25) purchased from Sigma Aldrich dispersed in isopropanol (IPA) to form a casting solution. Achieving a uniform, well-connected film required optimizing the TiO_2_:IPA ratio and dispersion technique. In preliminary tests, a 1:1 (wt:wt) TiO_2_–IPA mixture produced thick deposits that cracked upon drying, resulting in isolated domains with poor connectivity (Fig. 1**a**). Diluting the suspension (approximately 1:10 by weight) yielded a continuous granular film with no cracks (Fig. 1**b**). Ultrasonication of the TiO_2_ suspension for ∼10 min prior to deposition was employed to break up agglomerates and improve particle dispersion (Fig. 1**c**). Ultrasonication was performed in a heated water bath at 65°C, using the model Ironeside 506,482.

Each film was deposited through drop-casting ∼1–2 µL of the TiO_2_/IPA mixture into a channel on the sample holder (spanning the gap between electrodes) with a pipette. Drop-casting was performed in multiple small installments using a gentle rocking motion to spread the solution evenly. Films were air-dried for approximately 1 h at room temperature to evaporate the solvent, and this process was repeated 1–2 times to build up a thicker layer in a controlled manner. All samples were prepared and measured on the same day to avoid sedimentation or particle re-agglomeration. We found that these practices (using a high dilution ratio, sonication, and layered deposition) consistently produced TiO_2_ films that visually covered the electrode gap with minimal cracking.

The deposited TiO_2_ layers should therefore be regarded as irregular drop-cast particulate films with no precise control of film thickness beyond drop volume and total number of drops deposited. Film thickness was not independently measured by profilometry, AFM, or cross-sectional imaging in the present study, which limits any transport analysis based on absolute thickness. However, if the proposed methodology was combined with a profilometry measurement, it would further strengthen the possible analysis.

### Sample holder development through iterative prototyping (P1–P8)

**Fig. 2:**
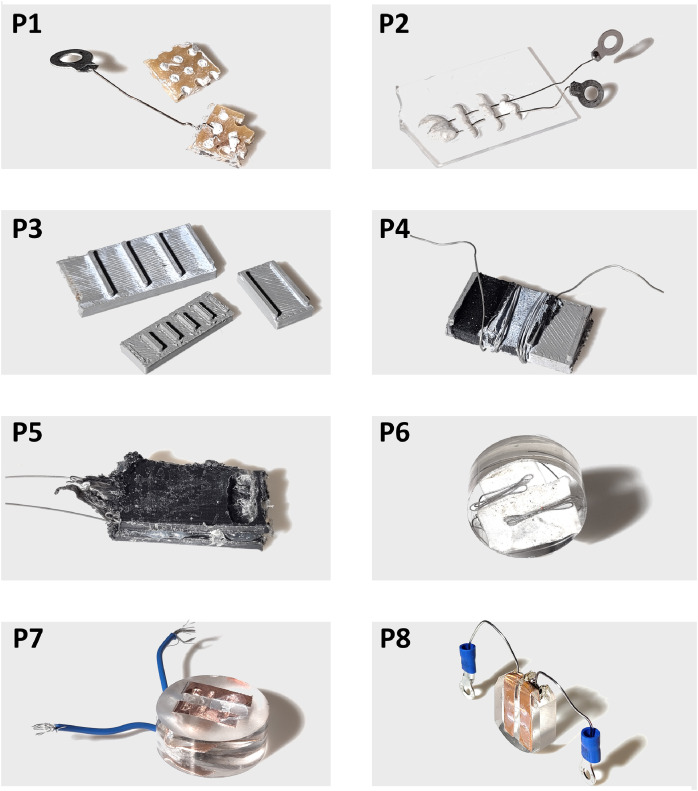
Iterations of sample holder prototypes (P1-P8).

Developing a reliable EBAC workflow for drop-cast thin films required the sample holder to solve two coupled problems: (i) spatial control of solvent/film placement and (ii) electrically clean, mechanically stable contacting compatible with the Point Electronic interface in a JEOL SEM. Across eight iterations (Fig. 2; Table 1), the design converged by removing failure modes that masked the film response (uncontrolled wetting, porous substrates, and parasitic conduction paths) and by improving contact robustness for repeated handling in the SEM chamber.

**Table 1:** Description of prototypes and observations for sample holder development.

Proto-type	Construction concept	Electrical contact concept	Main limitation observed	Design change introduced next
P1	Circuit board as substrate, pins integrated using adhesive gum	Point Electronic pins molded/held in adhesive gum	Poor control of solvent spreading; deposition area not repeatable	Add physical boundaries to constrain the solvent
P2	Glass substrate with adhesive gum “walls”	Pins located under/within gum structure	Solvent still wicked/spread along and around gum; insufficient containment	Move to structured (machined/printed) channels
P3	3D-printed substrates (two sizes), multiple channels for throughput	Mechanical contact points integrated in print	Material too porous; solvent absorbed/dispersed into the print; no containment	Seal/coat or switch materials; also consider conductive interface strategy
P4	3D-printed channels + conductive carbon adhesive layer	Wires (in addition to pins) for flexibility	Conductive carbon path dominated the electrical response; film contribution not observable	Remove highly conductive parasitic layers; embed conductors inside an insulating body
P5	“Lathed channel” concept: titanium wires between plastic plates	Embedded titanium wires as electrodes	Difficult to machine/drill evenly; channel depth/flatness was inconsistent leading to variable solvent behavior	Use castable body (resin) to improve machining quality and reproducibility
P6	Titanium wires embedded in a ClaroFast (resin/epoxy) pellet	Embedded wires intended as electrodes	Difficult to reliably access/contact the embedded wires externally	Replace wires with larger embedded conductors easier to expose and connect
P7	Bent copper plates embedded in ClaroFast	Copper exposed by engraving; solder on rigid wire	Channels had rounded edges and the solvent spread; rigid wire was mechanically fragile and broke during handling	Improve external contact robustness; use flexible (stranded) lead; adjust channel machining to expose copper + resin evenly
P8	Bent copper plates embedded in ClaroFast	Proper screw-pressed contact pads + flexible lead (stranded/solderable wire)		

The first concepts (P1–P2) prioritized fast interfacing with the Point Electronic pins using adhesive gum, but they failed to meet the core requirement of repeatable deposition geometry. On a circuit board substrate (P1), the solvent spread unpredictably across the surface; adding gum “walls” on a glass substrate (P2) provided *some* guidance but did not sufficiently contain the solvent, which still wicked around the gum boundaries. This motivated a shift toward proper physical channels rather than surface-defined paths.

Channel-based designs were explored next (P3–P4). Multi-channel 3D-printed holders (P3) introduced precise geometry, but the printed material proved too porous: solvent infiltrated the substrate, eliminating confinement and repeatability. A follow-up approach added a conductive carbon adhesive layer and switched to wire-based contacting (P4), but this introduced a new dominant artifact: the carbon layer's conductivity produced a strong parasitic signal that overwhelmed the electrical response of the deposited film. This established a key design rule for subsequent iterations: any auxiliary conductive material near the channel must not provide a low-resistance bypass path that dominates the measurement.

To avoid parasitic conduction while retaining defined channels, later iterations employed embedded electrodes within an insulating body with a machined deposition region (P5–P6). The first embedded-electrode concept used titanium wires between plastic plates with a mechanically cut channel (P5), but it was difficult to machine a flat, even channel to a controlled depth, leading to variable wetting and deposition thicknesses. Casting the electrode assembly into a resin pellet (P6) improved body rigidity and machinability, but it exposed another reproducibility bottleneck: reliable external electrical access to the embedded wires after casting.

### The final sample holder prototype (P8)

**Fig. 3:**
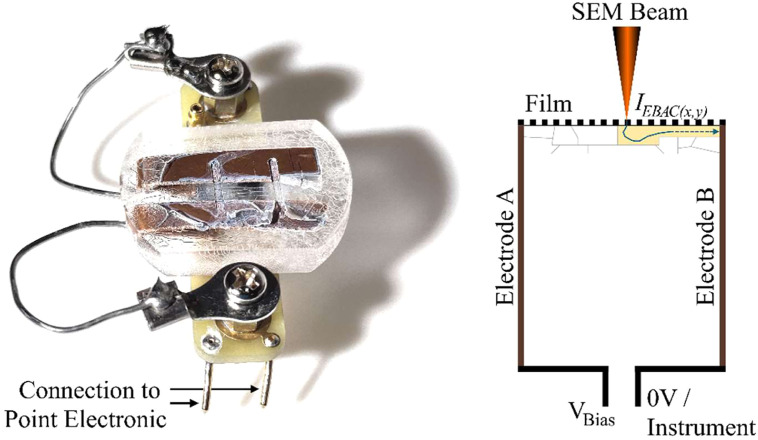
Left) P8 is shown connected to the Point Electronic Sample holder. Right) Simple schematic of the key components of the P8 functions in terms of signal acquisition and circuit.

The final prototype used the fundamental concepts from P5–P6, but it exchanged embedded wires with embedded copper plates (P7–P8), which are easier to expose and provide a larger contact area. In P7, copper plates were cast into ClaroFast (Struers, Ballerup, Denmark); the electrode was exposed by engraving; channels were formed with an engraving tool; and a wire was soldered on. While this solved access to the conductor, it still had two practical limitations: the channel geometry produced rounded edges, encouraging solvent spreading beyond the intended region, and the rigid soldered connection was mechanically fragile. It could break during handling in the SEM. The final design (P8) retained the copper-plate-in-resin design. Still, two improvements were made: (1) robust external connection via proper screw-pressed contact pads and a flexible stranded/solderable lead, and (2) repeatable channel formation using a 1 mm drill perpendicular to the surface to create a controlled geometry that exposes, in a single plane, both copper electrodes and the resin base at the channel bottom. This configuration enabled reproducible drop-casting into the channel and stable EBIC/conduction line scans dominated by the film rather than the holder. To facilitate mounting on the Point Electronic sample holder, the resin pellet was grounded flat on both sides. Fig. 3 shows P8 connected to the Point Electronic interface, with a simple schematic that illustrates the most important features of the final design. The mode of operation is illustrated, showing the absorbed electron path, moving through a domain of the film, and being collected at the electrode.

Silicon wafers with patterned electrodes would likely provide a flatter inter-electrode region and improved device-to-device uniformity. We chose the resin-and-copper approach because it allowed rapid prototyping, mechanical rework between iterations, and fabrication without cleanroom processing or photolithography. This lowered the barrier to iteration, which was an important practical advantage for fast prototyping, resulting in a sample holder that was more accessible for laboratories without access to a clean room.

### SEM and EBAC measurement procedure

EBAC measurements were carried out in a JEOL scanning electron microscope (JEOL SEM, model JSM-7200F) equipped with the Point Electronic EBIC module (Point Electronic GmbH). The SEM was operated in high-vacuum mode. Typically, an acceleration voltage of 5–15 keV was used for imaging and EBAC imaging, with beam currents of 2–18 nA. Lower beam energies (<5 keV) were tested to adjust the penetration depth in the TiO_2_ film, since shallower beams generate carriers closer to the top surface.

The measurement procedure was as follows:

1. **SEM imaging:** Secondary electron (SE) images were taken of the film and electrode region to examine the morphology and confirm that the film bridged the gap. This helped identify any obvious defects (cracks or uncovered areas).
2. **Line scans:** The electron beam scanned in a linear path across the sample (perpendicular to the electrode gap, crossing from one electrode, through the film, and to the other electrode) while the induced current was recorded. We repeated these line scans at different applied biases to observe the effects of electric field direction and magnitude. The scan speed was kept moderate to allow sufficient current averaging at each point, and multiple line scans (a minimum of three) were performed to ensure accurate measurements.
3. **2D mapping:** In some cases, we also performed a 2D raster scan of a region covering the gap and part of the electrodes. This produces an image where pixel intensity corresponds to the collected current at that beam position. Such maps can show the spatial distribution of conductivity or highlight specific current paths through the film.

During operation, care was taken to avoid beam-induced damage. High electron doses can potentially modify or degrade thin oxide films. We observed that especially line scans performed repeatedly at high beam voltage (>10 keV) could locally burn off the TiO_2_. Therefore, we limited the beam exposure and periodically checked the SE image for any visible changes to the film after scans. All data (line profiles and images) were recorded via the Point Electronic DISS5 system software.

## Method validation

To show that the workflow can distinguish an electrically open gap from a bridged thin film and produce a response with spatial information, we include two representative datasets: (1) EBAC maps acquired at three applied biases (0 V, 4 V, 9 V), and (2) line scans performed on the same holder geometry with and without a TiO_2_ film spanning the electrode gap.

### EBAC mapping (0 V, 4 V, 9 V)

**Fig. 4:**
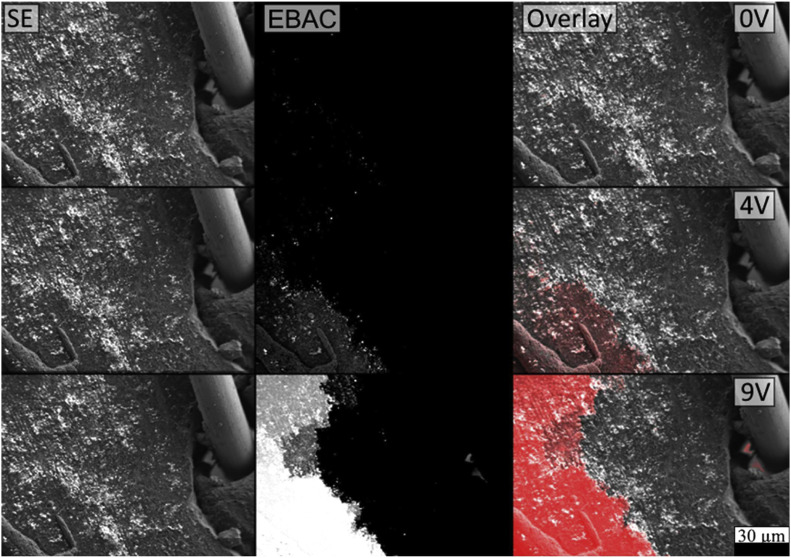
Secondary-electron (SE) images (left column), EBAC (middle column), and EBAC–SE overlays (right column) acquired at three applied biases (0 V, 4 V, 9 V). The overlay visualizes the EBAC signal (shown in red) on top of the SE morphology, where the red indicates the highly conductive zones.

Fig. 4 shows secondary-electron (SE) images, EBAC images, and an overlay where intensity is mapped onto the SE image. At 0 V, the EBAC signal is largely dark (near-background), indicating no measurable net current is collected under unbiased conditions. When an external bias is applied (4 V), a localized response becomes visible, and at 9 V the signal increases markedly and covers a larger fraction of the imaged region. This bias dependence supports the conclusion that the measured signal is linked to the applied electric field and to the presence of an electrically active pathway through the film area captured in the field of view.

### Line-scan control: no film vs TiO_2_ film

**Fig. 5:**
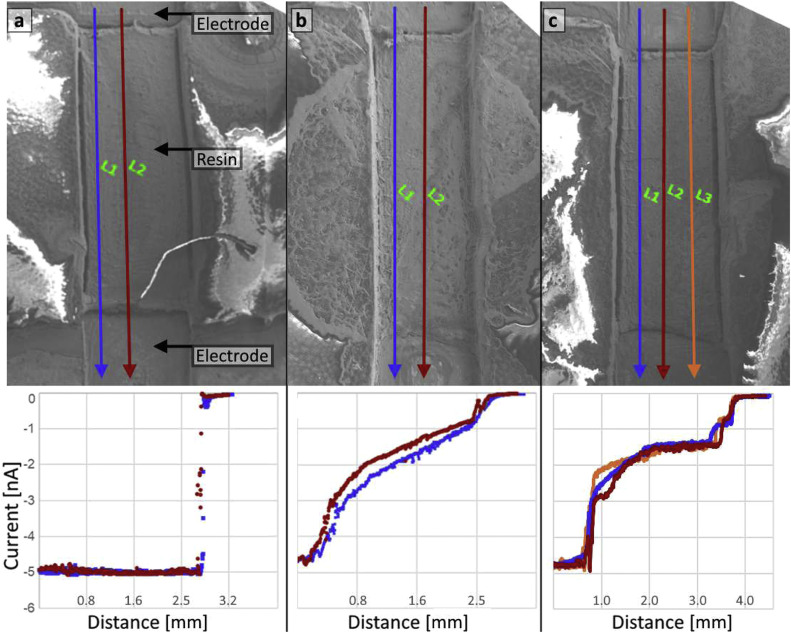
Top: SE images of the sample holder channel region with no film (a) and with a TiO_2_ film spanning the electrode gap (b and c). Two or three line-scan trajectories (L1, L2, and L3) are indicated (blue, red, and yellow arrows) and traverse the channel between the electrodes. Bottom: Corresponding current profiles (current in nA vs distance in mm). The no-film control shows a near-constant baseline across the channel, while the TiO_2_-film case shows a continuous position-dependent response across the scanned region.

Fig. 5 compares line scans recorded along two parallel trajectories (L1 and L2) across the channel region on (**a**) a holder with no film bridging the gap and (**b** and **c**) a holder with a TiO_2_ film bridging the electrodes. In the no-film case, the measured current remains near a constant baseline while scanning across the resin/channel region, with a pronounced change only when the beam approaches or crosses the metal electrode region. In contrast, when a TiO_2_ film is present, all line scans (L1, L2, and L3) show a clear, continuous position-dependent response across the scanned distance, rather than a flat baseline. The close agreement between different scans indicates that the signal trend is reproducible across nearby scan paths and is sensitive to whether the electrode gap is electrically bridged by the deposited film. The difference in signal between the empty and deposited sample holders can therefore be used to characterize the electrical properties of the thin film. As shown in the middle and right panels of Fig. 5, the signal changes depending on the inherent properties of the deposited film and variations that may come from drop-casting.

Together, these datasets demonstrate that the proposed holder design and data acquisition procedure generate a spatially resolved electrical response and can differentiate electrically disconnected regions (no-film control) from a continuous film spanning the electrode gap using both 2D EBAC mapping and 1D line profiling.

## Limitations

### Sample holder cleaning and reuse

The current cleaning protocol for removing deposited films (ultrasonication in isopropanol at ∼65°C) can degrade the ClaroFast resin over repeated cycles, leading to gradual disintegration of the holder body and reduced reproducibility. As a result, long-term reuse of the same holder across many trials is limited with this approach. A more robust cleaning strategy—ideally one that removes the TiO_2_ film without prolonged high-temperature sonication and without attacking the resin—would improve throughput and enable multi-trial reuse of a single holder.

### Channel geometry and solvent/film uniformity

The machined channel paths are not perfectly even across the full length of the deposition region. In particular, minor height/flatness differences between the exposed copper electrodes and the ClaroFast base can affect how the solvent wets the channel and how particles accumulate during drying. This can introduce variability in local film thickness, edge coverage, and ultimately signal uniformity. In the present study, we did not independently quantify the channel or film topography by AFM, stylus profilometry, or cross-sectional imaging. Future implementations should combine EBIC/EBAC mapping with independent topographic measurements and flatter electrode geometries.

### Interpretation of signals

While the method robustly produces line scans and maps sensitive to film continuity and gross nonuniformities, the quantitative meaning of the signal has not yet been fully established for these TiO_2_ films. In particular, the present configuration may probe a combination of conventional EBIC, electron-beam-absorbed-current (EBAC) / resistance-contrast behavior, local charging, contact resistance, beam-sample interaction volume, and field distribution. In the present work, we therefore interpret the measured contrast conservatively as a qualitative indicator of electrical connectivity between the beam position and the external electrodes, not as a standalone measurement of local conductivity or carrier-separation efficiency. However, with follow-up studies focused on calibrating measurements against established protocols and our proposed approach, the proposed sample holder may be a suitable tool for testing film continuity and electrical properties.

## Ethics statements

No human or animal studies were conducted. Experiments involved materials characterization in an SEM; all work was performed in accordance with institutional laboratory safety procedures for nanopowders and solvent handling.

## Related research article

None.

## CRediT authorship contribution statement

**Isaac Appelquist Løge:** Methodology, Software, Validation, Formal analysis, Investigation, Writing – original draft, Writing – review & editing, Funding acquisition. **Håkon Kristiansen:** Methodology, Validation, Writing – review & editing. **Nadia Shardt:** Formal analysis, Resources, Writing – review & editing, Supervision, Funding acquisition. **Tore Sandnes Vehus:** Conceptualization, Resources, Writing – review & editing, Supervision, Project administration, Funding acquisition.

## Declaration of competing interest

The authors declare that they have no known competing financial interests or personal relationships that could have appeared to influence the work reported in this paper.

## Data Availability

Data will be made available on request.
